# Conversion of DNA Copy Number Ratios to Mass Fractions for Quantifying Chicken Adulteration in Duck Meat by Digital PCR

**DOI:** 10.3390/foods15193523

**Published:** 2026-10-01

**Authors:** Sasithon Temisak, Jarupa Tunhyongnoppakoon, Jiranun Boonnil, Ganyarat Thaiwan, Chakkit Tanai, Watiporn Yenchum, Kanjana Hongthong, Nusara Yinyom, Phattaraporn Morris, Pattanapong Thangsunan

**Affiliations:** 1 Bioanalysis Group, Chemical Metrology and Biometry Department, National Institute of Metrology (NIMT), Pathumthani 12120, Thailand; sasithont@nimt.or.th (S.T.); jarupat@nimt.or.th (J.T.); jiranun@nimt.or.th (J.B.); watipon@nimt.or.th (W.Y.); kanjana@nimt.or.th (K.H.); nusaray@nimt.or.th (N.Y.); 2 Department of Biology, Faculty of Science, Naresuan University, Phitsanulok 65000, Thailand; ganyarat556677@gmail.com (G.T.); chakkit1380@gmail.com (C.T.); 3 Division of Biochemistry and Biochemical Innovation, Department of Chemistry, Faculty of Science, Chiang Mai University, Chiang Mai 50200, Thailand; 4 Materials Science Research Center, Faculty of Science, Chiang Mai University, Chiang Mai 50200, Thailand

**Keywords:** meat adulteration, chicken, duck, digital PCR, DNA copy number ratio, mass fraction

## Abstract

Meat adulteration, particularly the substitution of duck with chicken, threatens food authenticity, consumer trust, and regulatory compliance. However, converting DNA copy number measurements into meat mass fractions remains challenging. This study developed a digital PCR (dPCR)-based quantitative for determining chicken in duck. Singleplex and duplex dPCR assays targeting single-copy nuclear genes specific to chicken, duck and the conserved *myostatin* gene were developed and optimized. Five sets of binary chicken-in-duck mixtures were prepared and analyzed across 0–100% (*w*/*w*) to account for biological variability among meat sources. Five conversion approaches were evaluated: two conversion factor approaches based on the chicken-to-duck (*k_cd_*) and chicken-to-*myo* (*k_myo_*) copy number ratios, two corresponding approaches without conversion factors (*No_kcd_* and *Myo*), and a linear regression model (*Std*). Quantitative performance of all five conversion approaches was concentration-dependent, and no single approach was optimal across the entire range. Conversion factor approaches generally improved agreement with gravimetrically assigned values at low to intermediate levels but overestimated higher chicken fractions. The *Std* approach performed well at ≥5% (*w*/*w*) but was less reliable at lower levels. Approaches selected based on their quantitative performance were further evaluated across different sample compositions and processing conditions and applied to commercial duck products. Overall, dPCR combined with an appropriate conversion approach may support quantitative determination of chicken in duck meat for food authenticity testing.

## 1. Introduction

Meat adulteration in food products is considered a major concern in food safety and food authenticity. It affects public health and religious practices, causes financial loss, and reduces consumer confidence [1]. Meat adulteration refers to the intentional or unintentional substitution, mislabeling, or addition of undeclared meat components in food products. Generally, chicken is the cheapest type of meat and is therefore frequently used to substitute other higher-valued meats, particularly those with similar taste and texture, such as duck [2]. This problem is especially critical in fresh meat, minced meat, and processed meat products, where the meat species cannot be identified by visual inspection. As food product selection relies heavily on products’ description, the accuracy and reliability of the information provided on the labels are major concerns for consumers.

Recently, DNA-based methods have become essential tools for meat species adulteration. DNA can be considered a biological identification card due to its species-specificity. Moreover, unlike proteins, DNA remains relatively stable even after cooking or processing at high temperature and/or pressure, allowing reliable species identification and quantification in processed foods [3]. Common DNA-based molecular techniques applied to meat adulteration include conventional polymerase chain reaction (PCR), quantitative real-time PCR (qPCR), digital PCR (dPCR), sequencing, and related approaches [4,5,6,7]. Traditional PCR methods used for meat species identification often lack sufficient sensitivity, particularly when applied to processed or mixed meat products [8]. In contrast, qPCR, dPCR, and DNA sequencing can offer improved sensitivity, precision, and analytical reliability. Although qPCR has been widely adopted for meat species identification, it relies on a standard curve for quantitative measurement [9]. DNA sequencing analysis is also a powerful tool for meat species identification. However, their high cost, analytical complexity, and limited quantitative capability make it unsuitable for routine quantification compared with targeted approaches such as qPCR and dPCR [3,7].

Digital PCR can provide an absolute quantification by partitioning DNA into thousands of individual reactions, enabling direct counting of target molecules without the requirement of calibration curves and with correction for multiple target molecules per partition based on Poisson statistics [10]. This technique offers high precision and sensitivity, making it suitable for detecting and quantifying low levels of adulterants in meat mixtures [4]. A major challenge in DNA-based meat adulteration is translating molecular data, such as DNA copy number ratios, into practical metrics like mass fractions (*w*/*w*), which are more relevant for regulatory and consumer contexts. To address this challenge, single-copy numbers of nuclear DNA targets were selected. Unlike mitochondrial DNA, nuclear DNA exhibits stable copy numbers, thereby reducing one of the sources of variability when converting the DNA copy number ratio into a mass fraction [4,11,12].

Despite increasing application of dPCR for species detection, a scientifically validated framework for converting absolute DNA measurements into accurate mass-based estimates remains lacking. Many existing studies emphasize qualitative detection or apply a single empirical conversion factor without critically evaluating its assumptions, uncertainty, or applicability across matrices. Such simplified models may introduce bias because DNA yield, genome size, and tissue composition influence the relationship between measured DNA copies and the actual proportion of meat present. Therefore, our study aimed to develop singleplex and duplex droplet digital PCR (ddPCR) assays for the absolute quantification of chicken, duck, and *myostatin* (*myo*) targets and to investigate different strategies for converting DNA copy number measurements into meat mass fractions (*w*/*w*). Selected approaches were subsequently challenged using more complex multispecies mixtures and different processing conditions to assess their applicability beyond the binary model. Through this systematic evaluation, the study aimed to develop a quantitative framework for DNA-based determination of chicken in duck meat products, supporting improvements in the reliability and comparability of meat authenticity measurements.

## 2. Materials and Methods

### 2.1. Sample Preparation

Target meat species, including chicken and duck, were purchased as whole carcasses from local supermarkets. Non-target species—rat, Java dove, rabbit, ostrich, Japanese quail, and frog—were obtained by excising muscle tissue directly from the carcasses. Duck products, rice, and soy were purchased from local markets and supermarkets in Pathum Thani, Thailand. Reference materials—goat, crocodile, beef, pork, sheep, and salmon—were obtained from the National Institute of Metrology (NIMT, Pathum Thani, Thailand). Horse DNA was obtained from TUBITAK National Metrology Institute (UME, Kocaeli, Türkiye), and human genomic DNA was purchased from Promega (Madison, WI, USA). Whole raw chicken and duck meat from five individual samples of each species was processed separately in the form of biological replicates. For the preparation of the binary mixture, each sample was blended, freeze-dried (CHRIST, Gamma 1–16 LSC, Osterode am Harz, Germany), and stored at −80 °C until further use. Five independent sets (*N* = 5) of binary chicken-in-duck meat mixtures were prepared from these biological replicates at chicken mass fractions of 0%, 0.1%, 1%, 5%, 10%, 25%, 50%, 75%, 80%, and 100% (*w*/*w*). These mixtures were gravimetrically prepared using a six-digit analytical balance (Mettler Toledo, XPE26, Greifensee, Switzerland) to evaluate multiple approaches for converting DNA copy number measurements into meat mass fractions.

Additional sample sets were prepared for specific experimental evaluations. For the processing experiments, fresh samples were untreated raw meat. Boiled samples, representing cooked foods, were boiled for 10 min, whereas autoclaved samples, representing highly processed foods, were treated at 121 °C for 20 min. Pure chicken and duck samples in both freeze-dried and fresh meat were used to evaluate DNA extraction methods. Pure chicken and duck samples, together with binary mixtures containing 25% and 50% (*w*/*w*) of chicken in duck, were used to compare the singleplex and duplex ddPCR assays. To investigate the effects of the matrix on quantification, chicken-in-duck mixtures containing 10% and 75% (*w*/*w*) were subjected to three processing conditions: fresh, freeze-dried, and autoclaved. Mock multispecies mixtures were also prepared using different combinations of meat species and processing conditions. Their compositions and proportions are described in the following section. For each sample composition and concentration level, three independent portions (*n* = 3) were gravimetrically prepared by separate weighing using the analytical balance as described above.

### 2.2. DNA Extraction

To compare the variability among different DNA extraction methods, genomic DNA was extracted and purified using the DNeasy Mericon Food Kit, the Wizard Genomic DNA Purification Kit (Promega, WI, USA), the Salt Method [13], and the cetyltrimethylammonium bromide (CTAB) method combined with Genomic-tip 100/G (Qiagen, Hilden, Germany) according to the manufacturer’s protocol, using 0.2–1.0 g of sample. Each sample was extracted independently in triplicate (*n* = 3) as extraction replicates. The concentrations and purity of each extracted DNA were measured using a NanoDrop One Spectrophotometer (ThermoFisher, Waltham, MA, USA). Based on its low variability and suitability for quantitative analysis, the DNeasy Mericon Food Kit was selected for subsequent experiments.

### 2.3. Primers and Probes

The primers and probes used in this work were synthesized and purified by Integrated DNA Technologies (IDT, Singapore). The nucleotide sequences of the primers and probes, as well as the PCR product sizes, are presented in Table 1.

### 2.4. Quantitative Real-Time PCR and Droplet Digital PCR

In this study, qPCR was performed to evaluate assay specificity. Each reaction was carried out in a 20 µL volume containing 1X Luna Universal Probe qPCR Mix (New England Biolabs, MA, USA), 300 nM of each forward and reverse primer, 200 nM of probe, approximately 30 ng of DNA template, and nuclease-free water to volume. Amplification was conducted using a CFX96 real-time PCR system (Bio-Rad, Hercules, CA, USA) with the following thermal profile: an initial denaturation at 95 °C for 1 min, followed by 45 cycles of 95 °C for 15 s (denaturation) and 57 °C for 30 s (annealing/extension). qPCR data were analyzed using CFX Maestro software version 2.3 (Bio-Rad, Hercules, CA, USA). The ddPCR platform was used for absolute quantification of meat species. To perform ddPCR in a total volume of 20 µL, the reaction mixture contained 1X ddPCR SuperMix for probes (Bio-Rad, Hercules, CA, USA), 300 nM of each primer, 200 nM of probe, 5 U of *Hin*dIII (New England Biolabs, Ipswich, MA, USA), and the DNA template (approximately 1000 copies/µL or 5 ng/µL of sample). Duplex assays were performed by combining chicken–duck, chicken–*myo* or duck–*myo* primer–probe sets in a single reaction. The droplets were generated by AutoDG (Bio-Rad, Hercules, CA, USA). Thermal cycling was carried out on a thermal cycler (T100, Bio-Rad, Pleasanton, CA, USA) under the following conditions: 95 °C for 10 min, 40 cycles of 30 s at 94 °C, 1 min at 57 °C (optimal temperature), and 1 cycle at 98 °C for 10 min. To determine the optimal annealing temperature for ddPCR assays, the temperature gradient ranging from 55 to 65 °C was evaluated. After PCR amplification, the droplets were read using a QX200 Droplet Reader (Bio-Rad, Hercules, CA, USA), and data analysis was performed using QX Manager Software 2.1 Standard Edition (Bio-Rad, CA, USA). To determine DNA copy numbers, five biological sets of binary meat DNA samples were analyzed in duplicate, whereas the other samples were analyzed in triplicate as dPCR technical replicates.

### 2.5. Statistical Analysis

Grubbs’ test was used to identify potential outliers, and a two*-*tailed *t-*test was used to assess differences between the singleplex and duplex assays, with a significance level of α = 0.05. For each sample, copy number ratios were calculated separately for each replicate using the corresponding target copy number values. The resulting ratios were subsequently used to calculate the mean and standard deviation. Method performance was evaluated by calculating percent bias (%bias) to assess trueness, using the following equation: %bias = absolute (measured value − expected value)/expected value × 100. The coefficient of variation (%CV) was used to assess repeatability, calculated as follows: %CV = (SD/mean) × 100. Limits of detection (LODs) and limits of quantification (LOQs) were determined based on the variability and detection consistency of at least ten replicates at the lowest concentration levels, in accordance with the guideline provided in ISO 20395:2019 Biotechnology (Requirements for evaluating the performance of quantification methods for nucleic acid target sequences—qPCR and dPCR) [15]. The LOQ was defined as the lowest level meeting the predefined precision criterion (acceptance criterion for quantitative performance established at ≤25% CV and within 25% bias), while the LOD corresponded to the lowest level at which the target was reliably detected (≥95% detection rate, 95% CI), where detection rate = (number of positive results/total replicates) × 100 [16,17].

## 3. Results and Discussion

### 3.1. Specificity

To achieve accurate quantification of chicken in duck, nuclear DNA targets were selected in this study. Unlike mitochondrial DNA, nuclear DNA is located within the cell nucleus and typically shows stable and predictable copy numbers, with single-copy genes per haploid genome [18,19]. This characteristic makes nuclear DNA more appropriate for the quantitative analysis of meat adulteration. Previous studies have consistently demonstrated that a single-copy nuclear DNA target is better suited to qPCR via calibration curves and to dPCR for absolute quantification, allowing DNA copy number to be converted into mass fraction (*w*/*w*) [4,11,18]. The target species-specific genes for the detection of chicken, duck, and *myo* are shown in Table 1. All selected targets are single-copy genes and were adopted from the previous studies (Table 1). The specificity of the chicken, duck, and *myo* assays was evaluated using DNA samples extracted from different animal and plant species (Table 2). The chicken assay only detected DNA from chicken and showed no cross-reactivity with other avian species such as duck, Japanese quail, and Java dove, nor with non-avian animals and plants (Table 2). Similarly, the duck assay was highly specific to duck DNA and did not amplify DNA from any other tested species (Table 2). These results confirm the species-specificity of both the chicken and duck assays. The *myostatin* single-copy nuclear gene was also used as a reference assay to quantify total meat content in the samples, as this target gene is conserved across animal species. In this study, a *myo* assay by Griffiths’ group [12] was employed with slight modifications as the original *myo* assay [19] was unable to efficiently amplify duck DNA at the optimal annealing temperature of 57 °C used in both singleplex and duplex ddPCR assays (Figure S1B). The modified *myo* assay enabled the detection of not only mammals and avian species but also marsupials and reptiles [12]. This study also found that the modified *myo* assay did not amplify fish, frog, and plant-derived samples such as rice and soybean (Table 2). The *myostatin* is absent in plant-derived samples; therefore, these samples are not expected to produce a *myo*-specific signal. Nevertheless, they were included to evaluate potential signal interference in the assay.

### 3.2. Evaluation of DNA Extraction

**Figure 1:**
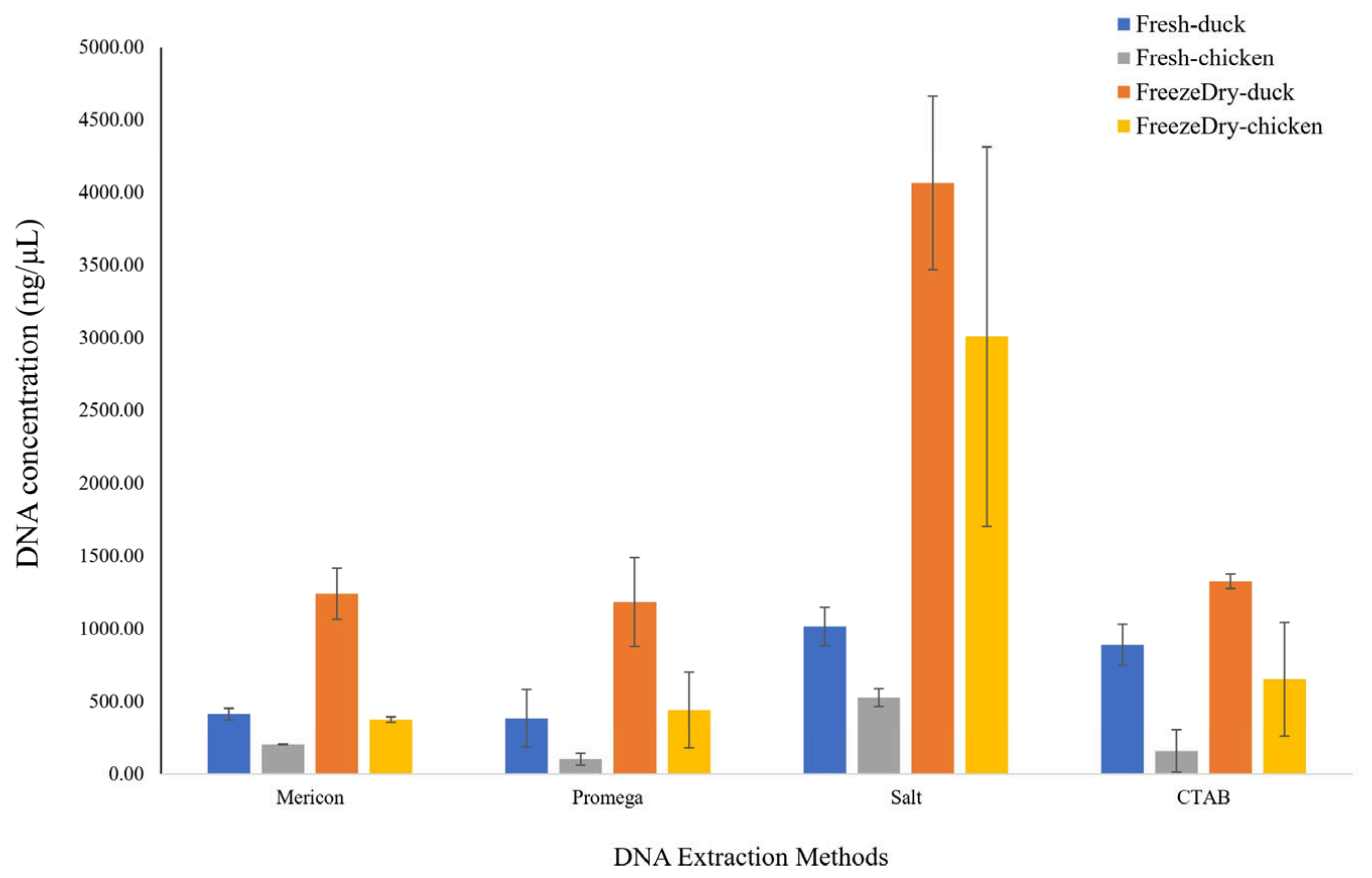
Comparison of DNA yields and standard deviations obtained from fresh and freeze-dried duck and chicken meat using different DNA extraction methods. Data are means ± SD.

Four DNA extraction methods were compared to to assess their suitability for quantitative analysis. For the same mass of meat, all extraction methods yielded higher DNA concentrations from duck than chicken (Figure 1). This may be because the density of duck cells is higher than that of chicken cells [20,21], resulting in a greater amount of duck DNA than chicken DNA at the same mass.

Although the salt method gave the highest DNA concentrations, the DNeasy Mericon Food Kit was selected for DNA preparation because it showed the lowest standard deviation (Figure 1). The DNeasy Mericon Food Kit provided consistent DNA yields and high-quality DNA (A260/280 ratios of 1.8–1.9 and A260/230 ratios of 1.8–2.0). According to each protocol, only the DNeasy Mericon Food Kit requires a fixed volume of lysate, whereas other methods rely on the entire lysate and require manual DNA precipitation steps, which increase the likelihood of DNA loss during processing, leading to higher variability in DNA yield. As this study focuses on quantitative metrology rather than maximizing DNA yield, precision and consistency were considered the determining criteria for method selection. After selecting the most repeatable method, the DNeasy Mericon Food Kit was applied to all study matrices. Moreover, using 200 mg (the minimum recommended input for this kit) of the sample provides sufficient DNA yield for qPCR and dPCR analysis.

### 3.3. Singleplex vs. Duplex Assays

**Figure 2:**
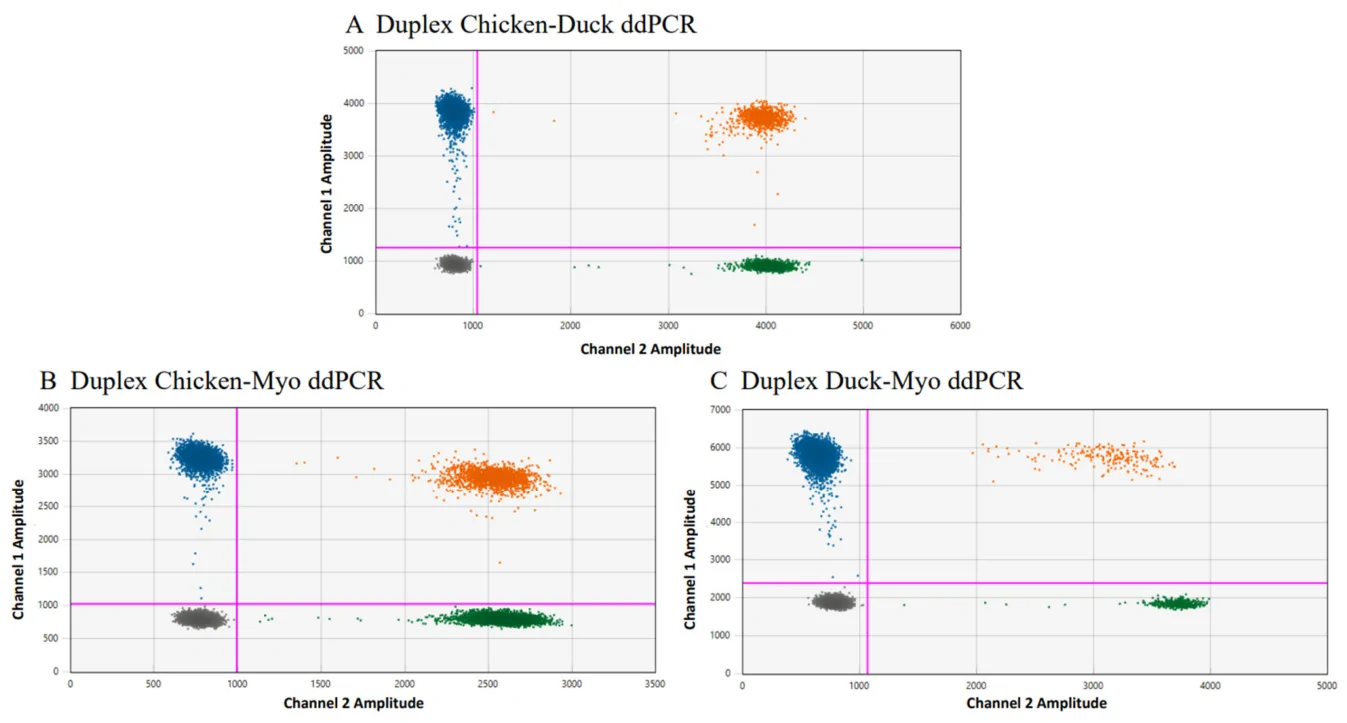
Two-dimensional plots for the optimization of the annealing temperature (57.0 °C) of the duplex assays for chicken–duck (**A**), chicken–*myo* (**B**) and duck–*myo* (**C**) using ddPCR. Black dot populations represent negative droplets; blue and green dot populations represent droplets positive for the respective target genes indicated in each panel; and orange dot populations represent droplets positive for both target genes.

The singleplex and duplex assays for duck, chicken, and *myo* were optimized on the ddPCR platform by evaluating a range of annealing temperatures. The optimal annealing temperature was selected based on the fluorescence amplitude separation between negative and positive droplet populations, the clarity of the droplet clusters, the absence of non-specific amplification, and minimal rain [4]. Based on these criteria, 57.0 °C was selected for the singleplex and duplex chicken, duck, and *myo* assays (Figure S2A and Figure 2 and Figure S2B–D, respectively) and was used for all subsequent ddPCR experiments.

**Figure 3:**
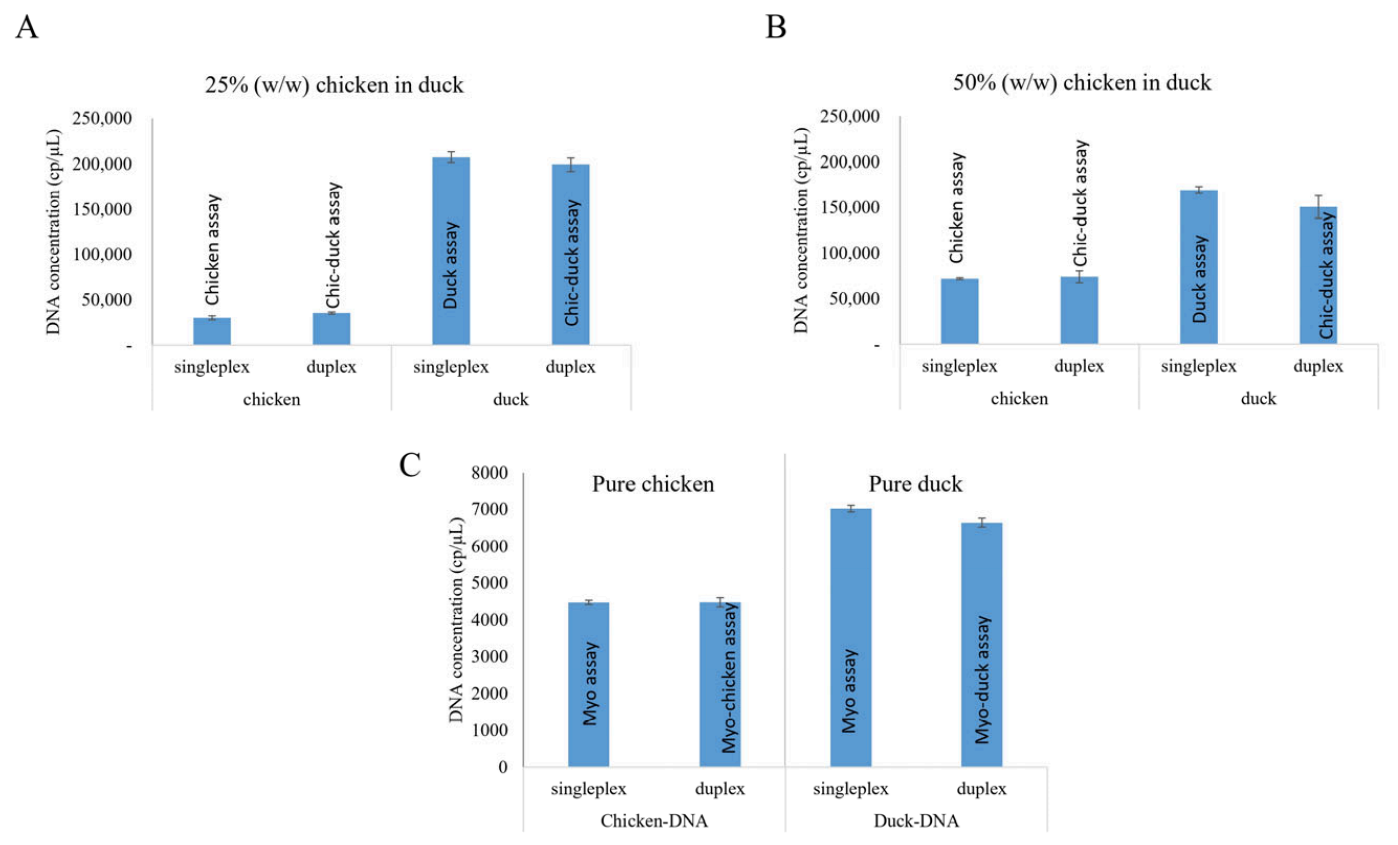
Comparison of DNA copy number (cp/µL) obtained by ddPCR using singleplex chicken, duck, and *myo* assays and duplex chicken–duck, chicken–*myo*, duck–*myo* assays in chicken and duck mixtures containing (**A**) 25%, (**B**) 50% (*w*/*w*), and (**C**) pure chicken and pure duck DNA. Data are means ± SD.

To evaluate between singleplex (chicken, duck, and *myo*) and duplex (chicken–duck, chicken–*myo*, and duck–*myo*) assays, DNA copy numbers of chicken, duck, and *myo* were compared using a chicken-in-duck meat mixture containing 0% (pure duck), 25%, 50%, 100% (pure chicken) (*w*/*w*). With the optimal condition, both singleplex and duplex ddPCR assays successfully quantified the target species and showed comparable results (*p* > 0.05) (Figure 3). These findings demonstrated that the duplex ddPCR assays were as reliable as the singleplex assays for simultaneous detection and quantification of chicken, duck, and *myo* in mixed meat samples. Duplex assays also reduce reagent consumption, consumable use, and analysis time [4,10]. Therefore, the duplex assays were selected for detecting and quantifying chicken, duck, or *myo*.

### 3.4. Mass Fraction Quantification by Conversion Approaches

As demonstrated in this study, the same meat mass yielded different concentrations of duck and chicken DNA (Figure 1 and Figure S3). Consequently, the copy number ratios of chicken and duck did not directly correspond to the mass fraction (*w*/*w*), despite the comparable genome sizes of the two species (approximately 1.1 Gb for duck, RefSeq accession no. GCA_000355885.1, and 1.1 Gb for chicken, RefSeq accession no. GCA_000002315.1) [22,23,24]. To further investigate whether this observation could be attributed to variability among species-specific assays, two different assays for duck DNA quantification were compared: one targeting the *prolactin* gene [12] and the other targeting chromosome 1 [25]. No significant difference was observed between the two duck assays (Figure S1A). Furthermore, when the *myo* assay was used as an internal control and reference gene, the copy number ratios of chicken or duck to *myo* were close to or equal to one in pure chicken or pure duck DNA samples, even across different DNA concentrations (Figure S1C).

To further minimize the potential bias arising from matrix effects and water content, the freeze-dried meats were also used, and the water content of chicken and duck was investigated. The results showed that there were no differences between the two meats, with water content of approximately 3% (*w*/*w*), as measured by coulometric Karl Fischer titration (Mettler Toledo, Switzerland). Collectively, these results indicate that the DNA copy number ratio between duck and chicken does not directly reflect the mass ratios. As a result, a conversion factor may be required.

To investigate this, approaches with and without a *k* constant were proposed to convert DNA copy number fractions into mass fractions;

(1) *k_cd_* constant, based on the ratio of chicken to the chicken and duck DNA, was determined using Equation (1).

(1) $$\frac{M_{chic}}{\left(M_{chic}+M_{duck}\right)}=k_{cd}\times \frac{Q_{chic}}{\left(Q_{chic}+Q_{duck}\right)}$$

(2) The no-*k_cd_* approach (*No_kcd_*) applied Equation (1) without *k_cd_* using Equation (2).

(2) $$\frac{M_{chic}}{\left(M_{chic}+M_{duck}\right)}=\frac{Q_{chic}}{\left(Q_{chic}+Q_{duck}\right)}$$

(3) *k_myo_* constant, based on *myostatin* gene to quantify total meat content, was calculated using Equation (2).

(3) $$\frac{M_{chic}}{M_{toltal}}=k_{myo}\times \frac{Q_{chic}}{Q_{myo}}$$

(4) The no-*k_myo_* approach (*Myo*) applied Equation (3) without *k_myo_* using Equation (4).

(4) $$\frac{M_{chic}}{M_{toltal}}=\frac{Q_{chic}}{Q_{myo}}$$

where

$M_{chic}$ is the chicken weight by balance;

$M_{duck}$ is the duck weight by balance;

$M_{total}$ is the duck and duck weight by balance;

$Q_{chic}$ is the chicken DNA copy number;

$Q_{duck}$ is the duck DNA copy number;

$Q_{myo}$ is the *myo* DNA copy number.

(5) The linear regression-based conversion approach (*Std*) used standard curve-based linear correction to convert the observed DNA copy number ratio (y) to the corresponding mass fraction (x) using a linear calibration model (y = ax + b), where y represents the percentage of chicken DNA calculated as the chicken DNA copy number divided by the sum of chicken and duck DNA copy numbers obtained by dPCR and multiplied by 100; the slope (a) represents the conversion factor between x and y; and the intercept (b) accounts for the baseline response (Figure S4).

**Figure 4:**
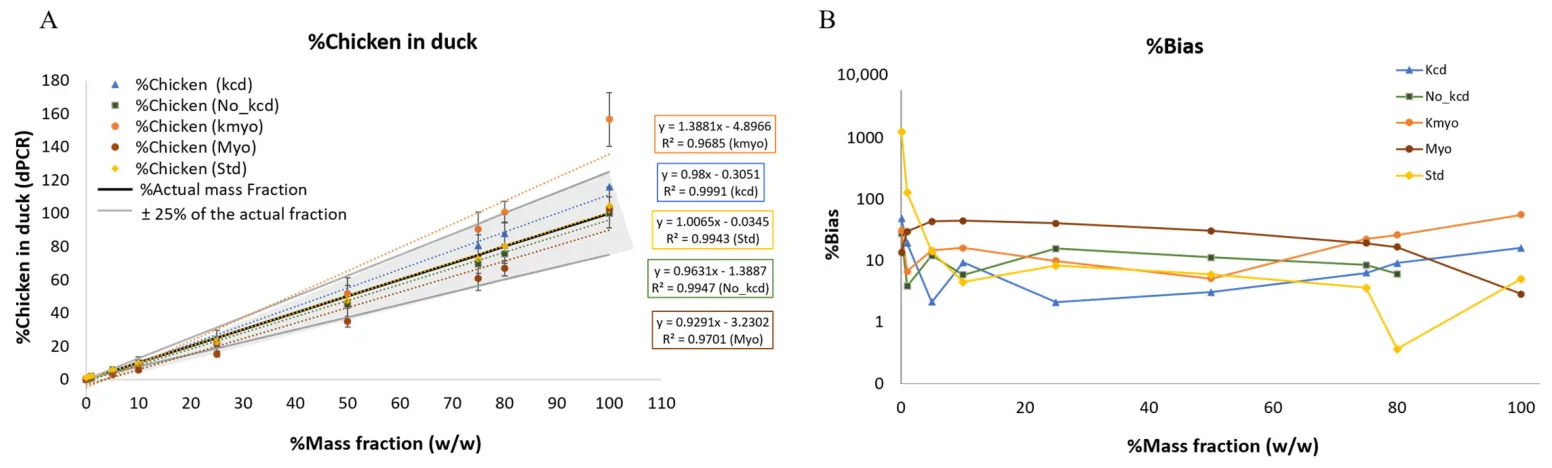
Comparison of the four conversion approaches for converting DNA copy number ratios into chicken mass fractions in chicken–duck mixtures: (**A**) estimated chicken mass fractions across the working range (the gray shaded area represents ±25% relative deviation from the gravimetric mass fraction); and (**B**) the corresponding percentage %bias.

The *k_cd_* and *k_myo_* conversion factors, calculated from five biological replicates using Equations (1) and (3), were 1.16 ± 0.28 and 1.51 ± 0.12 (mean ± SD), respectively. The five proposed approaches were then applied to estimate chicken mass fractions from DNA copy numbers (Table 3, Figure 4A). Overall, different DNA-to-mass fraction conversion approaches showed different performance depending on the chicken mass fraction.

Without the *k_cd_* constant (*No_kcd_*), the copy number ratios agreed well with the gravimetric values over most of the investigated range, although higher variability was observed at 5% and 10% chicken, with CVs of 39.88% and 26.85%, respectively. Application of the *k_cd_* conversion factor improved the agreement at intermediate chicken levels (5–50% *w*/*w*), reducing the relative bias to approximately 2–9% (Figure 4B). However, this improvement was not observed across the entire range; greater positive bias occurred at 0.1% and at high chicken fractions, reaching 16.05% for pure chicken (Table 3, Figure 4B).

Without the *k_myo_* constant (*Myo*), the approach showed good precision across the investigated range but generally underestimated the chicken mass fraction in mixed samples, particularly at 5–50% (*w*/*w*), where relative biases ranged from approximately 30–44% (Table 3). Application of the *k_myo_* conversion factor substantially improved the agreement with the gravimetric values at 1–50% chicken, reducing the relative bias to approximately 5–16% (Figure 4B). At higher chicken fractions, however, the *k_myo_* increasingly overestimated the chicken content, with biases of 26.10% and 55.35% at 80% and 100%, respectively.

The *Std* approach showed a different performance profile, providing low bias and good precision at chicken levels ≥5% (*w*/*w*), with relative biases below 15% and CVs below 14% (Table 3). In contrast, poor performance was observed at 0.1–1%, with high positive biases (127–1235%) and CVs of 73–137% (Table 3, Figure 4B). An apparent positive result was also obtained for the 0% chicken sample. Thus, although matrix-matched calibration provided reliable quantitative results at intermediate to high chicken levels, its application at low levels was limited under the conditions investigated and required preparation of appropriate calibration standards.

Overall, no single approach provided optimal performance across the entire concentration range. The *No_kcd_* approach offered a simple conversion-factor-free strategy for binary chicken–duck mixtures, while applying *k_cd_* generally improved quantitative agreement at intermediate chicken levels. Similarly, the direct *Myo* approach tended to underestimate chicken content, whereas *k_myo_* improved agreement at low to intermediate levels but increasingly overestimated it at higher fractions. These findings indicate that conversion factors can improve the agreement between DNA copy number ratios and gravimetric mass fractions over specific concentration ranges, but a single conversion factor may not provide optimal correction across the entire 0.1–100% range. The *Std* approach performed well at ≥5% chicken, although its limited performance at trace levels and the need for matched calibration standards may restrict its practical application. As expected, application of the fixed *k* factors had no effect on %CV, indicating that the conversion factors primarily affected the estimated mass fraction rather than measurement precision.

Due to the limitation of the six-digit gravimetric balance (Mettler Toledo) used for preparing 1 g mixed-meat samples, the lowest achievable weight level was 0.1% (*w*/*w*) chicken in duck. When applying the developed approaches, the LOD of all methods was 0.1% (*w*/*w*), with a 100% detection rate. The LOQ obtained from the developed approaches was also 0.1% (*w*/*w*) for the *Myo* approach; 1% (*w*/*w*) for the *k_cd_*, *No_kcd_*, and *k_myo_*; and 5% (*w*/*w*) for the *Std* approach [15,16].

### 3.5. Examination of Freeze-Dried, Fresh, and Processed Meat Samples

**Figure 5:**
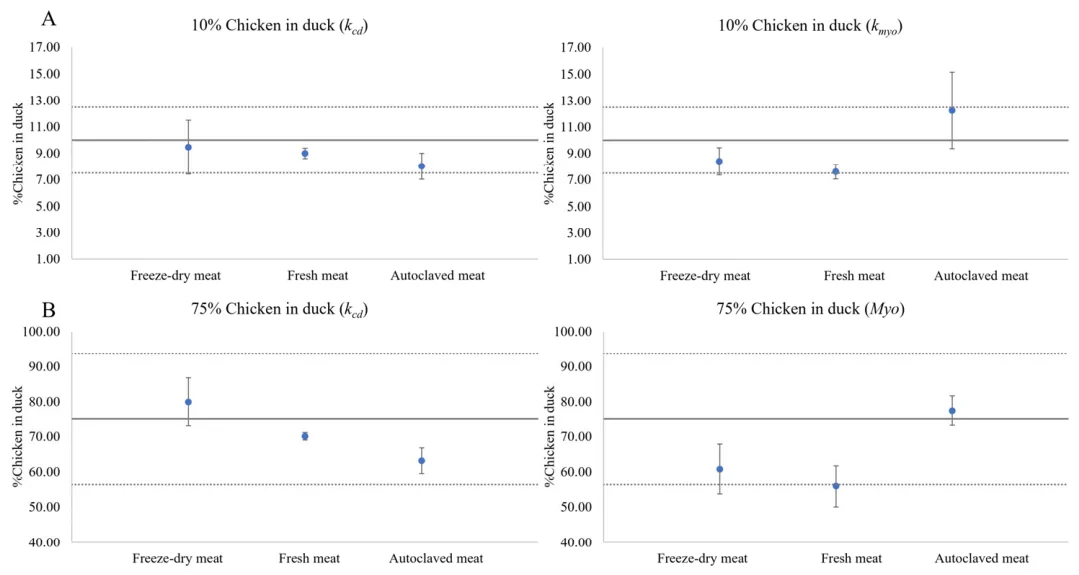
Measured percentage of chicken in duck meat mixtures across different meat processing treatments: freeze-dried, fresh, and autoclaved treatment. Data are means ± SD. The solid horizontal line indicates the true value: (**A**) 10% mass fraction of chicken in duck and (**B**) 75% mass fraction of chicken in duck. Dotted lines represent ±25% relative deviation thresholds (i.e., the acceptance criterion).

To investigate the effect of processing conditions on quantification, chicken-in-duck mixtures containing 10% and 75% (*w*/*w*) chicken were evaluated under three conditions: freeze-dried, fresh, and autoclaved. Based on their quantitative performance, the *k_cd_* and *k_myo_* approaches were selected for the 10% chicken level, whereas the *k_cd_* and *Myo* approaches were chosen for the 75% chicken level. These selected approaches provided mass fraction values within the predefined acceptance criterion (±25%) [11] relative to the target proportions (Figure 5). These results indicate that the selected DNA-to-mass fraction approaches maintained acceptable quantitative performance across freeze-dried, fresh, and autoclaved samples, supporting their applicability under different processing conditions.

### 3.6. Transition from a Binary Model System to Multispecies Application

The conversion approaches were initially established and evaluated using binary chicken–duck mixtures (Section 3.4). Among the approaches investigated, the *k_cd_* and *k_myo_* approaches were selected for further evaluation because they represent two distinct conversion strategies: *k_cd_* is based on the relative abundance of chicken and duck species-specific targets, whereas *k_myo_* relates the chicken-specific target to the *myostatin* target as an indicator of total meat-derived DNA. Their contrasting denominator concepts provided an opportunity to examine how the conversion factors established in binary mixtures would perform when additional meat species are present. Accordingly, the *k_cd_* and *k_myo_* approaches were further evaluated using mock multispecies samples containing chicken, duck, pork, ostrich and turkey at different proportions and under different processing conditions, including freeze-dried, fresh, boiled, and autoclaved samples (Table 4).

Chicken, duck, and *myo*-specific DNA targets were successfully detected and quantified in all mock mixtures across the different processing conditions (Table 4; mock samples). Notably, the DNA yield extracted from autoclaved samples was lower than that obtained from other meat matrices, likely due to extensive DNA degradation caused by severe thermal processing. Despite this reduction in yield, the remaining DNA fragments were still amplifiable by ddPCR. This is because thermal treatment fragments genomic DNA into smaller pieces that remain suitable for amplification of the short amplicons (typically 70–200 bp) used in qPCR and dPCR assays [26]. Moreover, in ddPCR workflows, a restriction enzyme is often included to digest high-molecular-weight genomic DNA to improve template dispersion and ensure accurate partitioning of target molecules among droplets for absolute quantification [27]. This step becomes less critical when analyzing highly processed or heat-treated samples, where DNA fragmentation has already occurred. However, extensive DNA degradation may still affect quantification if the assay target region is disrupted.

Both the *k_cd_* and *k_myo_* approaches produced estimated chicken mass fractions (mean ± SD) within ±25% relative deviation from the target proportions for the tested mock samples (Table 4 and Figure S5). Overall, the *k_cd_* approach provided estimates that were generally closer to the assigned chicken mass fractions than those obtained using the *k_myo_* approach, particularly for the boiled samples containing turkey. Nevertheless, both approaches showed acceptable quantitative performance across the multispecies compositions and processing conditions evaluated in this study. These findings extend the results obtained with binary chicken–duck mixtures (Section 3.4) and support the potential applicability of both approaches to more complex meat mixtures, while also indicating that their performance may depend on species composition and processing conditions. Further studies with larger sample sizes and other meat species that may realistically occur in duck products may be required to refine the scope and assess their practical applicability. Moreover, although *myostatin* was used as a common nuclear DNA reference in meat, potential variation in the *myostatin* among species and breeds may affect *myo*-based quantification and should be further evaluated.

### 3.7. Challenge with the Real Samples

Commercial duck products were subsequently analyzed to assess the applicability of the methods (Table 4). All commercial products were labeled “100% duck”. The results showed no detectable chicken DNA in duck sausage, dumplings, frozen duck parts, duck minced meat from Supermarket Brand 1, roasted duck, and five-spice braised duck (Table 4; commercial samples). Trace levels of chicken DNA were detected in three products consisting of duck minced meat from the local market, duck minced meat from Supermarket Brand 2 and sun-dried duck (Table 4; commercial samples). Duck minced meat from the local market and sun-dried duck contained low levels of chicken. The estimated chicken mass fractions were below the LOQ of 1% for both the *k_cd_* and *k_myo_* approaches, whereas the *Myo* approach, with an LOQ of 0.1%, yielded quantifiable values of 0.5 ± 0.2% and 0.2 ± 0.1% (*w*/*w*), respectively (Table 4). These low levels may be attributed to incidental cross-contamination during processing, equipment sharing, or retail handling rather than deliberate adulteration.

Notably, in duck minced meat from Supermarket Brand 2, the combined copy number of the chicken and duck (2.8 × 10^5^ cp/µL) was within the mean ± SD range of the *myo* copy number (2.7 × 10^5^ cp/µL ± 7.5 × 10^4^ cp/µL), supporting the assumption that the measured meat-derived DNA could be adequately represented as a binary mixture of chicken and duck. This sample, Supermarket Brand 2, exhibited a markedly higher chicken proportion (approximately 46.1% ± 19.2% with the *k_cd_* and 62.2% ± 27.6% with the *k_myo_*) (Table 4; commercial samples). The detected level greatly exceeds what would be expected from accidental contamination and may indicate undeclared addition of chicken meat or substantial mixing during production. In contrast, for some products, including duck sausage, dumpling, and five-spice braised duck, the combined copy numbers of the chicken and duck were outside the mean ± SD range of the *myo* copy number (Table 4; commercial samples). These values may suggest the presence of other meat species not targeted in chicken and duck.

## 4. Conclusions

In this study, we developed and evaluated dPCR-based approaches for the quantitative determination of chicken in duck meat products by combining species-specific targets with the *myostatin* gene as a common meat reference. Five approaches for converting DNA copy number ratios to mass fractions (*w*/*w*) were evaluated, highlighting the importance of selecting an appropriate DNA-to-mass conversion strategy. Among the approaches evaluated, the use of conversion factors improved quantitative agreement within specific concentration ranges, although no single approach provided optimal performance across the entire 0.1–100% (*w*/*w*) range.

The selected approaches were further evaluated under different processing conditions and in mock multispecies mixtures, where they generally provided acceptable quantitative performance. Application to commercial duck products showed the potential to detect undeclared chicken. However, quantitative results for unknown samples should be interpreted with caution, particularly when they fall below the LOQ, where detection should be regarded as target detection rather than a reliable quantitative estimate.

Overall, this study demonstrated the importance of appropriate DNA-to-mass conversion when translating DNA copy number measurements into gravimetric meat mass fractions. Such conversion is particularly relevant because food composition and labeling are commonly expressed on a mass basis. Combining dPCR with an appropriate DNA-to-mass conversion approach may therefore provide a useful basis for the quantitative assessment of meat composition and support food authenticity testing. Further evaluation across a broader range of meat species and product matrices would help assess the potential application of these approaches to routine food authenticity testing.

## Figures and Tables

**Table 1:** Primer and probe sequences used for the detection of chicken, duck, and *myostatin* targets in this work.

Target Species	Primer/Probe Name	Oligonucleotide Sequence (5′ to 3′)	Target	Length (bp)	Ref
Chicken	Chicken_F	CAGCTGGCCTGCCGGC	TGF- *β*3 gene	77	[14]
	Chicken_R	GCCCAGTGGAATGTGGTATTCA			
	Chicken_P-FAM	TGCCACTCCTCTGCACCCAGTGC			
Duck	Duck_F	GAGATGTTCAAGAAAATAAAGCTGT	*Prolactin receptor* gene	91	[12]
	Duck_R	CTCTCACTGTTAGAAAGGAGTG			
	Duck_P_Hex	HEX/TG GGA AAC T/ZEN/C AGT GTG TAG CCT CAG AAC GG/IABkFQ/			
*Myostatin*	Myo_F	TTGTGCARATCCTGAGACTCAT	*Myostatin* gene	77	[12]
	Myo_R	GTCAAGTTTCARAGATCGRATT			
	Myo_Hex	HEX/CC CAT GAA A/ZEN/G AYG GTA CAA GRT ATA CTG/IABkFQ/			

**Table 2:** Specificity testing of chicken, duck, and *myo* assays across various animal and plant species.

Sample	Chicken Assay *	Duck Assay *	*Myo* Assay *
Chicken (*Gallus gallus*)	24.78 ± 1.20	Not detected	26.18 ± 0.64
Duck (*Anas platyrhynchos)*	Not detected	25.32 ± 0.36	27.57 ± 0.14
Goat (*Capra hircus*)	Not detected	Not detected	25.77 ± 0.07
Crocodile (*Crocodylus siamensis*)	Not detected	Not detected	25.88 ± 0.11
Beef (*Bos taurus*)	Not detected	Not detected	20.18 ± 0.16
Pork (*Sus scrofa*)	Not detected	Not detected	26.27 ± 0.11
Rat (*Rattus argentiventer*)	Not detected	Not detected	25.78 ± 0.23
Horse (*Equus ferus*)	Not detected	Not detected	26.11 ± 0.07
Sheep (*Ovis aries*)	Not detected	Not detected	25.93 ± 0.04
Java dove (*Streptopelia risoria*)	Not detected	Not detected	23.76 ± 0.07
Rabbit (*Oryctolagus cuniculus*)	Not detected	Not detected	27.04 ± 0.17
Japanese quail (*Coturnix japonica*)	Not detected	Not detected	23.06 ± 0.03
Ostrich (*Struthio camelus*)	Not detected	Not detected	26.25 ± 0.17
Turkey (*Meleagris gallopavo*)	Not detected	Not detected	25.25 ± 0.08
Human (*Homo sapiens*)	Not detected	Not detected	24.46 ± 2.02
Salmon (*Salmo salar*)	Not detected	Not detected	Not detected
Frog (*Hoplobatrachus rugulosus*)	Not detected	Not detected	Not detected
Rice (*Oryza sativa*)	Not detected	Not detected	Not detected
Soya (*Glycine max*)	Not detected	Not detected	Not detected

* Dectected results are representd as Cq values (mean ± SD).

**Table 3:** Conversion of DNA copy number ratios to mass fractions using different proposed approaches.

%Chicken in Duck—Mass Fraction (*w*/*w*)	%Chicken (*k_cd_*)	%Bias	%CV	%Chicken (*No_kcd_*)	%Bias	%CV	%Chicken (*k_myo_*)	%Bias	%CV	%Chicken (*Myo*)	%Bias	%CV	%Chicken (*Std*)	%Bias	%CV
0	0.00	NA	NA	0.00	NA	NA	0.00	NA	NA	0.00	NA	NA	1.19	NA	156.35
0.1	0.15	47.97	22.38	0.13	27.50	22.38	0.13	30.60	16.53	0.09	9.69	16.53	1.33	1234.74	137.18
1	1.19	19.14	24.01	1.04	3.84	24.01	1.07	6.63	16.33	0.71	29.42	16.33	2.27	126.72	73.34
5	5.11	2.10	39.88	4.40	12.02	39.88	4.27	14.52	15.13	2.83	43.42	15.13	5.70	14.04	13.26
10	10.92	9.24	26.85	9.41	5.87	26.85	8.40	16.01	12.29	5.56	44.40	12.29	9.56	4.44	13.23
25	24.48	2.08	22.30	21.09	15.63	22.30	22.53	9.89	9.10	14.91	40.35	9.10	22.94	8.24	10.34
50	51.53	3.06	18.10	44.40	11.19	18.10	52.53	5.05	9.41	34.77	30.46	9.41	47.03	5.94	10.63
75	79.72	6.29	8.62	68.69	8.41	8.62	91.79	22.38	11.73	60.76	18.99	11.73	72.30	3.60	2.59
80	87.21	9.02	8.35	75.15	6.06	8.35	100.88	26.10	6.86	66.78	16.53	6.86	80.29	0.36	1.28
100	116.05	16.05	0.00	100.00	0.00	0.00	155.35	55.35	11.48	102.84	2.84	11.48	105.00	5.00	5.69

All data were obtained from *N* = 5 (independent meat sources; 5 individual chickens and 5 individual ducks), 3 independent DNA extractions per mixture, and ddPCR duplicate = 2 (technical replicates).

**Table 4:** Determination of %chicken in samples using the *k_myo_* and *Nor* approaches to convert DNA copy numbers by ddPCR into mass fractions.

Samples	Description	Identification Testing Results (Mean cp/µL ± SD)			Quantification Testing Results (Mean ± SD)	
		*Myo*	*Chicken*	*Duck*	*%Chicken (* *k_cd_)*	*%Chicken (k* *_myo_)*
Mock samples						
Mixed meat (Freeze-dried)	chicken 5%, duck 50%, pork 45%	3.3 × 10^5^ ± 2.8 × 10^4^	1.4 × 10^4^ ± 1.6 × 10^3^	2.9 × 10^5^ ± 1.2 × 10^4^	5.5% ± 1.4%	6.6% ± 0.8%
Mixed meat (Freeze-dried)	chicken 10%, duck 45%, pork 45%	2.9 × 10^5^ ± 1.9 × 10^4^	2.4 × 10^4^ ± 4.3 × 10^3^	2.1 × 10^5^ ± 2.6 × 10^4^	11.9% ± 1.5%	12.6% ± 3.1%
Mixed meat (Freeze-dried)	chicken 50%, duck 25%, pork 25%	2.6 × 10^5^ ± 1.7 × 10^4^	9.5 × 10^4^ ± 9.9 × 10^3^	1.0 × 10^5^ ± 9.2 × 10^3^	55.5% ± 4.6%	55.8% ± 9.5%
Mixed meat (Fresh)	chicken 1%, duck 50%, ostrich 49%	1.0 × 10^5^ ± 1.8 × 10^4^	1.1 × 10^3^ ± 5.5 × 10^2^	8.0 × 10^4^ ± 1.3 × 10^3^	1.7% ± 0.9%	1.8% ± 1.0%
Mixed meat (Boiled)	chicken 1%, duck 50%, ostrich 49%	3.0 × 10^5^ ± 6.8 × 10^4^	1.7 × 10^3^ ± 3.7 × 10^2^	1.6 × 10^5^ ± 4.5 × 10^4^	1.2% ± 0.3%	0.9% ± 0.2%
Mixed meat (Boiled)	chicken 5%, duck 50%, turkey 45%	2.8 × 10^5^ ± 4.8 × 10^4^	6.4 × 10^4^ ± 2.8 × 10^3^	1.1 × 10^5^ ± 1.7 × 10^4^	6.6% ± 3.6%	3.8% ± 2.3%
Mixed meat (Boiled)	chicken 10%, duck 45%, turkey 45%	2.0 × 10^5^ ± 2.7 × 10^4^	7.9 × 10^3^ ± 1.7 × 10^3^	7.7 × 10^4^ ± 1.0 × 10^4^	10.9% ± 2.4%	6.0% ± 1.6%
Mixed meat (Autoclaved)	chicken 1%, duck 50%, ostrich 49%	2.9 × 10^4^ ± 8.3 × 10^3^	1.6 × 10^2^ ± 4.5 × 10	2.2 × 10^4^ ± 7.2 × 10^3^	0.9 ± 0.3%	0.9% ± 0.2%
Mixed meat (Autoclaved)	chicken 5%, duck 50%, ostrich 45%	2.9 × 10^4^ ± 1.1 × 10^3^	1.3 × 10^3^ ± 6.0 × 10^2^	2.8 × 10^4^ ± 8.1 × 10^3^	4.8% ± 1.2%	6.6% ± 1.9%
Mixed meat (Autoclaved)	chicken 10%, duck 45%, ostrich 45%	2.8× 10^4^ ± 4.9 × 10^3^	2.5 × 10^3^ ± 6.0 × 10^2^	2.3 × 10^4^ ± 3.4 × 10^3^	11.2% ± 2.1%	13.3% ± 2.0%
Commercial samples	Labeled					
Duck sausage (Cooked)	duck 100%	4.2 × 10^4^ ± 2.1 × 10^3^	0	2.7 × 10^4^ ± 3.2 × 10^3^	0%	0%
Duck dumpling (Cooked)	duck 100%	1.5 × 10^5^ ± 2.6 × 10^3^	0	1.0 × 10^5^ ± 4.9 × 10^3^	0%	0%
Frozen duck parts (Fresh)	duck 100%	1.3 × 10^5^ ± 4.0 × 10^4^	0	1.2 × 10^5^ ± 3.3 × 10^4^	0%	0%
Duck minced (Market, fresh)	duck 100%	1.4 × 10^5^ ± 2.5 × 10^4^	6.8 × 10^2^ ± 2.8 × 10^2^	1.1 × 10^5^ ± 2.4 × 10^4^	0.6% ± 0.2%	0.7% ± 0.2%
Duck minced (Supermarket, fresh)-1	duck 100%	8.5 × 10^4^ ± 2.7 × 10^3^	0	7.8 × 10^4^ ± 2.4 × 10^4^	0%	0%
Duck minced (Supermarket, fresh)-2	duck meat 65%, duck gizzard 35%	2.7 × 10^5^ ± 7.5 × 10^4^	1.1 × 10^5^ ± 5.0 × 10^4^	1.7 × 10^5^ ± 5.5 × 10^4^	46.1% ± 19.2%	62.2% ± 27.6%
Roasted Duck (Cooked)	duck 100%	1.2 × 10^5^ ± 2.5 × 10^4^	0	9.2 × 10^4^ ± 3.8 × 10^4^	0%	0%
Five-spice braised duck (Cooked)	duck 100%	6.0 × 10^5^ ± 1.2 × 10^5^	0	3.4 × 10^5^ ± 4.8 × 10^4^	0%	0%
Sun-dried duck (Cooked)	duck 100%	4.7 × 10^4^ ± 2.8 × 10^3^	6.8 × 10 ± 3.6 × 10	3.7 × 10^4^ ± 1.8 × 10^4^	0.2% ± 0.1%	0.3% ± 0.1%

*n* = 3 independent DNA extractions, with technical triplicates using ddPCR.

## Data Availability

The data presented in this study are available on request from the corresponding authors.

## Supplementary Materials

The following supporting information can be downloaded at: https://www.mdpi.com/article/10.3390/foods15193523/s1, Figure S1: Comparison of ddPCR results: (A) quantification of duck DNA by different duck-specific assays, one from Griffiths et al. [12] and the other from ISO/TS-20224-10 [25]; (B) comparison of two *myo* assays, one from Laube et al. [19] and the other from Griffiths et al. [12]; and (C) quantification of duck, chicken and *myo* target using duplex ddPCR with duck–*myo* and chicken–*myo* assays. A ten-fold dilution series of purified duck and chicken DNA was used. The left y-axis represents copy number concentration, and the right y-axis represents the copy number ratio of the target gene to the *myo* gene. The error bars are SD. Figure S2: Optimization of annealing temperature on the ddPCR platform using chicken and duck DNA: (A) singleplex ddPCR of chicken, duck, and *myo* assays; (B) duplex chicken–duck assay; (C) duplex chicken–myo assay; (D) duplex duck–*myo* assay. Figure S3: Copy numbers of duck and chicken DNA obtained by singleplex dPCR using DNA isolated from 200 g of freeze-dried duck and chicken with the DNeasy Mericon Food Kit (Qiagen, Germany). Data are presented as means ± SD. Figure S4: Example of a standard curve of %chicken in duck generated by the observed values against mass fraction values and used to derive the equation for converting DNA copy number ratios into mass fractions. Data are presented as means ± SD. Figure S5: %Chicken in multispecies samples, converted by the *k_myo_* and *k_cd_* approaches across different meat processing treatments (freeze-dried, fresh, boiled, and autoclaved treatment). Data are means ± SD. The solid horizontal line indicates the true value from the mass fraction of chicken, while dotted lines represent ±25% relative deviation thresholds.
