# Supplementary material for: Conversion of DNA Copy Number Ratios to Mass Fractions for Quantifying Chicken Adulteration in Duck Meat by Digital PCR
Source: Foods. 2026 Oct 1;15(19):3523. doi: 10.3390/foods15193523 (PMC13665225; doi:10.3390/foods15193523)
Supplement: Supplementary file 1 [file foods-15-03523-s001.zip › foods-4553134-supplementary.pdf]

Supplementary Materials

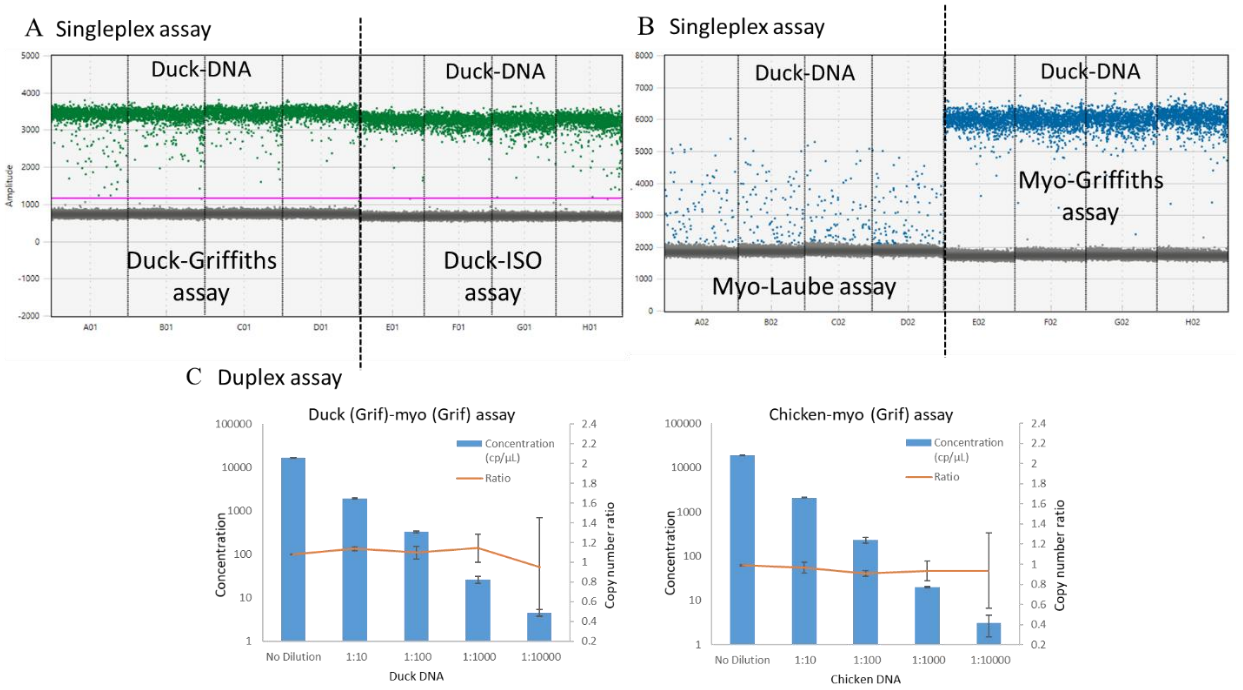

Figure S1. Comparison of ddPCR results: (A) quantification of duck DNA by different duck-specific assays, one from Griffiths et al. [12] and the other from ISO/TS-20224-10 [25]; (B) comparison of two *myo* assays, one from Laube et al. [19] and the other from Griffiths et al. [12]; and (C) quantification of duck, chicken and *myo* target using duplex ddPCR with duck-*myo* and chicken-*myo* assays. A ten-fold dilution series of purified duck and chicken DNA was used. The left y-axis represents copy number concentration and the right y-axis represents the copy number ratio of target gene to *myo* gene. The error bars are SD.

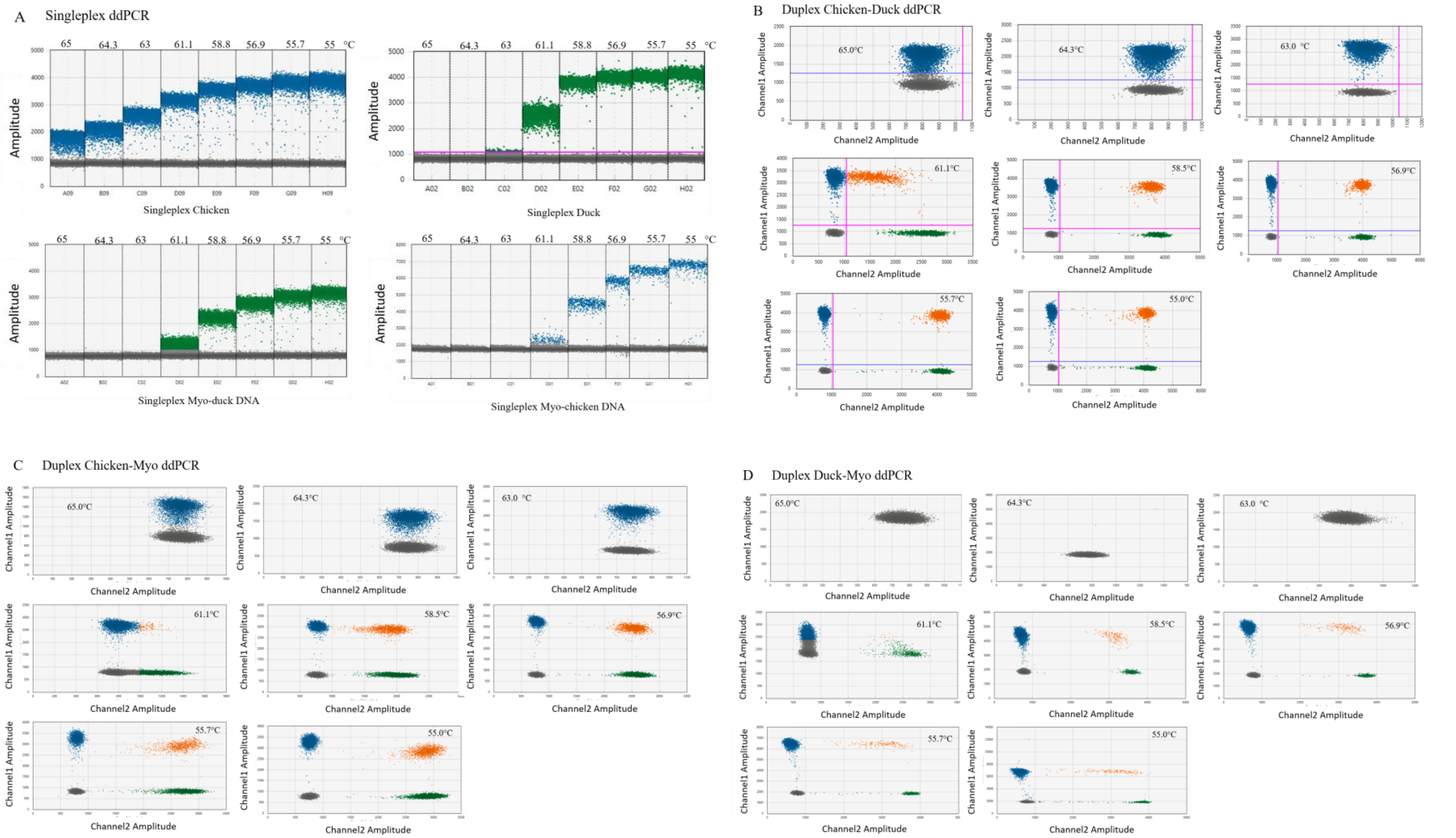

Figure S2. Optimization of annealing temperature on the ddPCR platform using chicken and duck DNA: (A) singleplex ddPCR of chicken, duck, and *myo* assays; (B) duplex chicken-duck assay; (C) duplex chicken-*myo* assay; (D) duplex duck-*myo* assay.

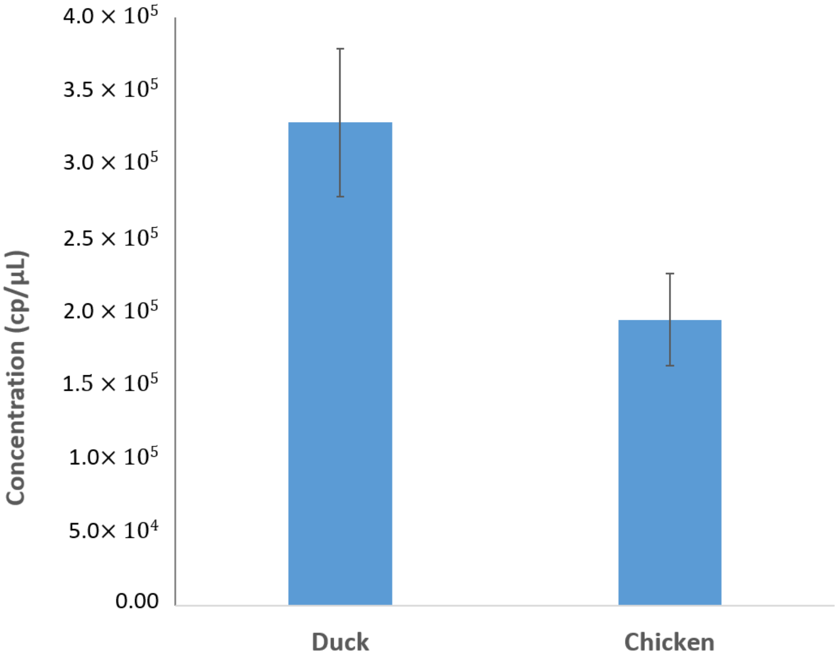

Figure S3. Copy number of duck and chicken DNA obtained by singleplex dPCR using DNA isolated from 200 g of freeze-dried duck and chicken DNeasy Mericon Food Kit (Qiagen, Germany). Data are presented as means ± SD.

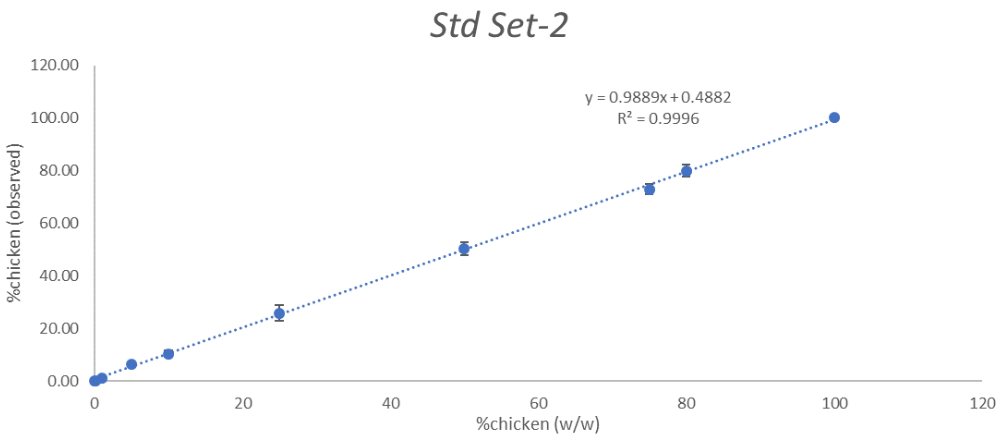

Figure S4. The example of standard curve of %chicken in duck generated by the observed values against mass fraction values, used to derive the equation for converting DNA copy number ratio into mass fractions. Data are presented as means ± SD.

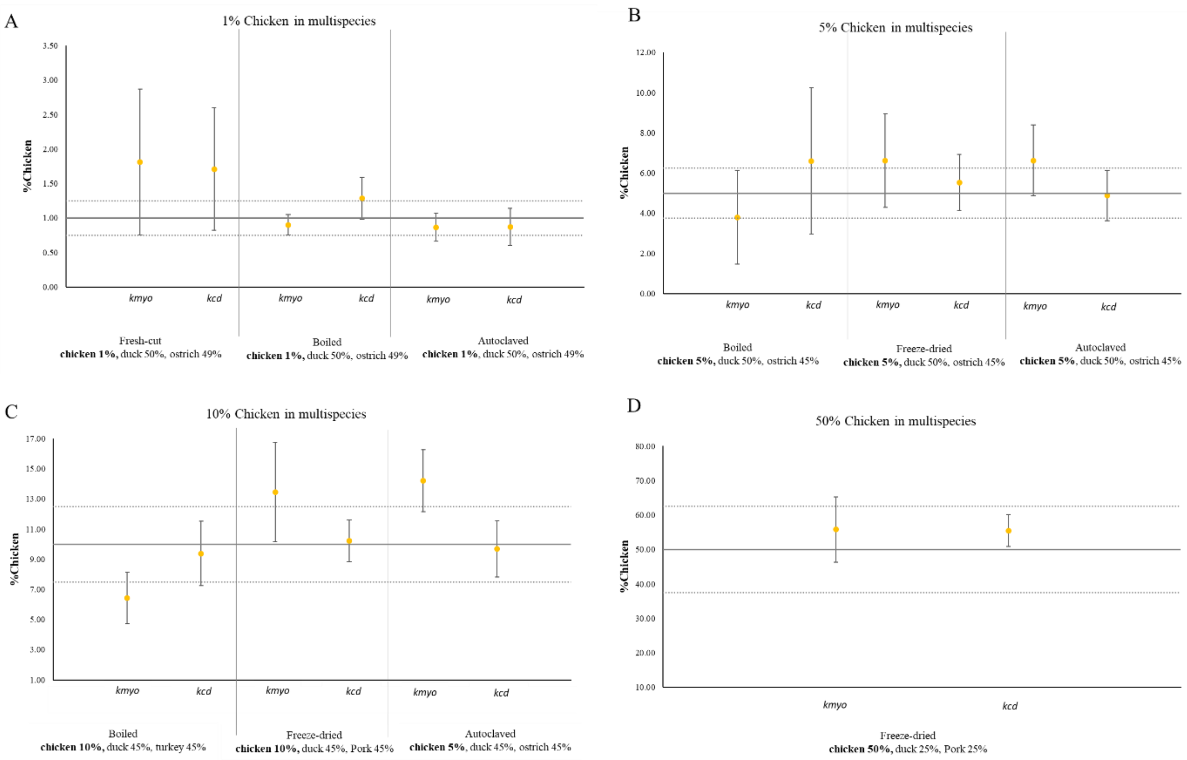

Figure S5. %Chicken in multispecies converting by k_{myo} and k_{cd} approaches across different meat processing treatments: freeze-dried, fresh, boiled and autoclaved treatment. Data are means ± SD. The solid horizontal line indicates the true value from mass fraction of chicken, while dotted lines represent ±25% relative deviation thresholds.
